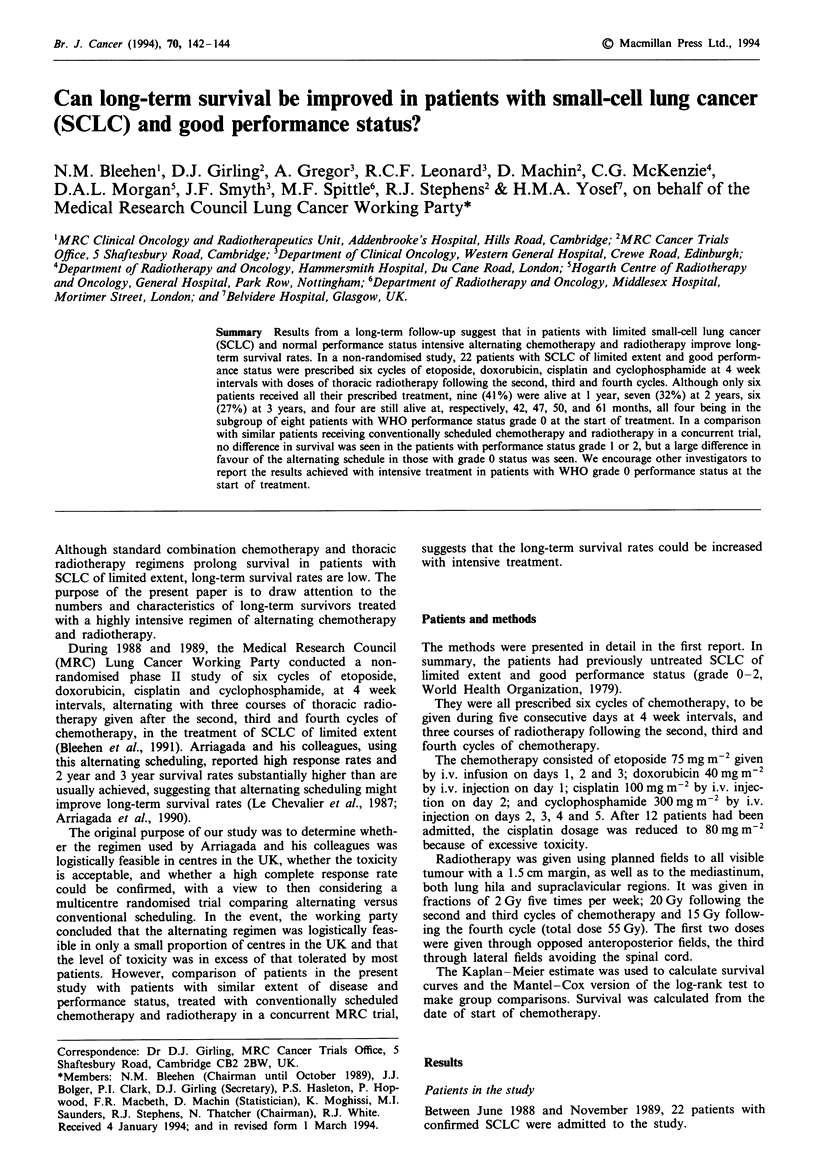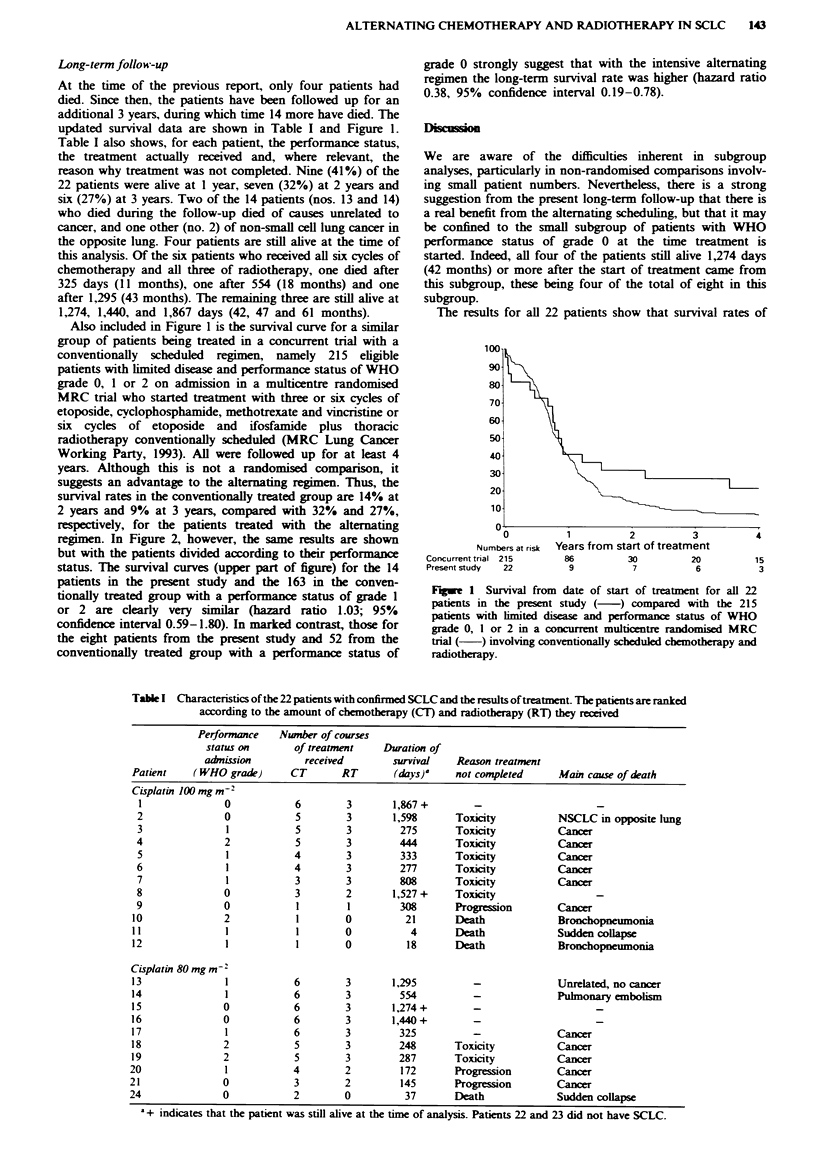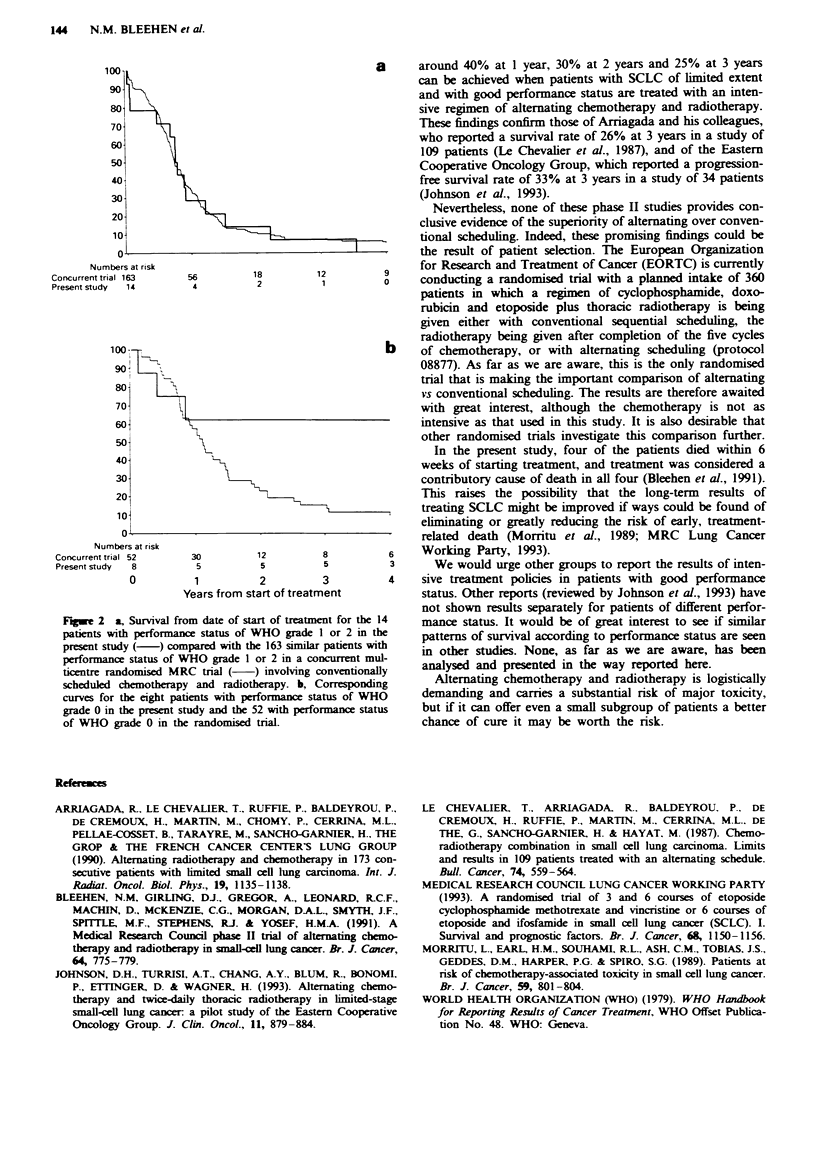# Can long-term survival be improved in patients with small-cell lung cancer (SCLC) and good performance status? Medical Research Council Lung Cancer Working Party.

**DOI:** 10.1038/bjc.1994.264

**Published:** 1994-07

**Authors:** N. M. Bleehen, D. J. Girling, A. Gregor, R. C. Leonard, D. Machin, C. G. McKenzie, D. A. Morgan, J. F. Smyth, M. F. Spittle, R. J. Stephens

**Affiliations:** MRC Clinical Oncology and Radiotherapeutics Unit, Addenbrooke's Hospital, Cambridge, UK.

## Abstract

Results from a long-term follow-up suggest that in patients with limited small-cell lung cancer (SCLC) and normal performance status intensive alternating chemotherapy and radiotherapy improve long-term survival rates. In a non-randomised study, 22 patients with SCLC of limited extent and good performance status were prescribed six cycles of etoposide, doxorubicin, cisplatin and cyclophosphamide at 4 week intervals with doses of thoracic radiotherapy following the second, third and fourth cycles. Although only six patients received all their prescribed treatment, nine (41%) were alive at 1 year, seven (32%) at 2 years, six (27%) at 3 years, and four are still alive at, respectively, 42, 47, 50, and 61 months, all four being in the subgroup of eight patients with WHO performance status grade 0 at the start of treatment. In a comparison with similar patients receiving conventionally scheduled chemotherapy and radiotherapy in a concurrent trial, no difference in survival was seen in the patients with performance status grade 1 or 2, but a large difference in favour of the alternating schedule in those with grade 0 status was seen. We encourage other investigators to report the results achieved with intensive treatment in patients with WHO grade 0 performance status at the start of treatment.


Br. J. Cancer (1994), 70, 142-144 © Macmillan Press Ltd., 1994

### Can long-term survival be improved in patients with small-cell lung cancer (SCLC) and good performance status?

N.M. Bleehen[1], D.J. Girling[2], A. Gregor[3], R.C.F. Leonard[3], D. Machin[2], C.G. McKenzie[4], D.A.L. Morgan[5], J.F. Smyth[3], M.F. Spittle[6], R.J. Stephens[2] & H.M.A. Yosef[7], on behalf of the Medical Research Council Lung Cancer Working Party*

[1]*MRC Clinical Oncology and Radiotherapeutics Unit, Addenbrooke's Hospital, Hills Road, Cambridge;* [2]*MRC Cancer Trials Office, 5 Shaftesbury Road, Cambridge;* [3]*Department of Clinical Oncology, Western General Hospital, Crewe Road, Edinburgh;* [4]*Department of Radiotherapy and Oncology, Hammersmith Hospital, Du Cane Road, London;* [5]*Hogarth Centre of Radiotherapy and Oncology, General Hospital, Park Row, Nottingham;* [6]*Department of Radiotherapy and Oncology, Middlesex Hospital, Mortimer Street, London; and* [7]*Belvidere Hospital, Glasgow, UK.*

**Summary** Results from a long-term follow-up suggest that in patients with limited small-cell lung cancer (SCLC) and normal performance status intensive alternating chemotherapy and radiotherapy improve long-term survival rates. In a non-randomised study, 22 patients with SCLC of limited extent and good performance status were prescribed six cycles of etoposide, doxorubicin, cisplatin and cyclophosphamide at 4 week intervals with doses of thoracic radiotherapy following the second, third and fourth cycles. Although only six patients received all their prescribed treatment, nine (41%) were alive at 1 year, seven (32%) at 2 years, six (27%) at 3 years, and four are still alive at, respectively, 42, 47, 50, and 61 months, all four being in the subgroup of eight patients with WHO performance status grade 0 at the start of treatment. In a comparison with similar patients receiving conventionally scheduled chemotherapy and radiotherapy in a concurrent trial, no difference in survival was seen in the patients with performance status grade 1 or 2, but a large difference in favour of the alternating schedule in those with grade 0 status was seen. We encourage other investigators to report the results achieved with intensive treatment in patients with WHO grade 0 performance status at the start of treatment.

Although standard combination chemotherapy and thoracic radiotherapy regimens prolong survival in patients with SCLC of limited extent, long-term survival rates are low. The purpose of the present paper is to draw attention to the numbers and characteristics of long-term survivors treated with a highly intensive regimen of alternating chemotherapy and radiotherapy.

During 1988 and 1989, the Medical Research Council (MRC) Lung Cancer Working Party conducted a non-randomised phase II study of six cycles of etoposide, doxorubicin, cisplatin and cyclophosphamide, at 4 week intervals, alternating with three courses of thoracic radiotherapy given after the second, third and fourth cycles of chemotherapy, in the treatment of SCLC of limited extent (Bleehen *et al.*, 1991). Arriagada and his colleagues, using this alternating scheduling, reported high response rates and 2 year and 3 year survival rates substantially higher than are usually achieved, suggesting that alternating scheduling might improve long-term survival rates (Le Chevalier *et al.*, 1987; Arriagada *et al.*, 1990).

The original purpose of our study was to determine whether the regimen used by Arriagada and his colleagues was logistically feasible in centres in the UK, whether the toxicity is acceptable, and whether a high complete response rate could be confirmed, with a view to then considering a multicentre randomised trial comparing alternating versus conventional scheduling. In the event, the working party concluded that the alternating regimen was logistically feasible in only a small proportion of centres in the UK and that the level of toxicity was in excess of that tolerated by most patients. However, comparison of patients in the present study with patients with similar extent of disease and performance status, treated with conventionally scheduled chemotherapy and radiotherapy in a concurrent MRC trial, suggests that the long-term survival rates could be increased with intensive treatment.

### Patients and methods

The methods were presented in detail in the first report. In summary, the patients had previously untreated SCLC of limited extent and good performance status (grade 0-2, World Health Organization, 1979).

They were all prescribed six cycles of chemotherapy, to be given during five consecutive days at 4 week intervals, and three courses of radiotherapy following the second, third and fourth cycles of chemotherapy.

The chemotherapy consisted of etoposide 75 $mg\,m^{-2}$ given by i.v. infusion on days 1, 2 and 3; doxorubicin 40 $mg\,m^{-2}$ by i.v. injection on day 1; cisplatin 100 $mg\,m^{-2}$ by i.v. injection on day 2; and cyclophosphamide 300 $mg\,m^{-2}$ by i.v. injection on days 2, 3, 4 and 5. After 12 patients had been admitted, the cisplatin dosage was reduced to 80 $mg\,m^{-2}$ because of excessive toxicity.

Radiotherapy was given using planned fields to all visible tumour with a 1.5 cm margin, as well as to the mediastinum, both lung hila and supraclavicular regions. It was given in fractions of 2 Gy five times per week; 20 Gy following the second and third cycles of chemotherapy and 15 Gy following the fourth cycle (total dose 55 Gy). The first two doses were given through opposed anteroposterior fields, the third through lateral fields avoiding the spinal cord.

The Kaplan-Meier estimate was used to calculate survival curves and the Mantel-Cox version of the log-rank test to make group comparisons. Survival was calculated from the date of start of chemotherapy.

### Results

### *Patients in the study*

Between June 1988 and November 1989, 22 patients with confirmed SCLC were admitted to the study.

Correspondence: Dr D.J. Girling, MRC Cancer Trials Office, 5 Shaftesbury Road, Cambridge CB2 2BW, UK.
*Members: N.M. Bleehen (Chairman until October 1989), J.J. Bolger, P.I. Clark, D.J. Girling (Secretary), P.S. Hasleton, P. Hopwood, F.R. Macbeth, D. Machin (Statistician), K. Moghissi, M.I. Saunders, R.J. Stephens, N. Thatcher (Chairman), R.J. White.
Received 4 January 1994; and in revised form 1 March 1994.

*Long-term follow-up*

At the time of the previous report, only four patients had died. Since then, the patients have been followed up for an additional 3 years, during which time 14 more have died. The updated survival data are shown in Table I and Figure 1. Table I also shows, for each patient, the performance status, the treatment actually received and, where relevant, the reason why treatment was not completed. Nine (41%) of the 22 patients were alive at 1 year, seven (32%) at 2 years and six (27%) at 3 years. Two of the 14 patients (nos. 13 and 14) who died during the follow-up died of causes unrelated to cancer, and one other (no. 2) of non-small cell lung cancer in the opposite lung. Four patients are still alive at the time of this analysis. Of the six patients who received all six cycles of chemotherapy and all three of radiotherapy, one died after 325 days (11 months), one after 554 (18 months) and one after 1,295 (43 months). The remaining three are still alive at 1,274, 1,440, and 1,867 days (42, 47 and 61 months).

Also included in Figure 1 is the survival curve for a similar group of patients being treated in a concurrent trial with a conventionally scheduled regimen, namely 215 eligible patients with limited disease and performance status of WHO grade 0, 1 or 2 on admission in a multicentre randomised MRC trial who started treatment with three or six cycles of etoposide, cyclophosphamide, methotrexate and vincristine or six cycles of etoposide and ifosfamide plus thoracic radiotherapy conventionally scheduled (MRC Lung Cancer Working Party, 1993). All were followed up for at least 4 years. Although this is not a randomised comparison, it suggests an advantage to the alternating regimen. Thus, the survival rates in the conventionally treated group are 14% at 2 years and 9% at 3 years, compared with 32% and 27%, respectively, for the patients treated with the alternating regimen. In Figure 2, however, the same results are shown but with the patients divided according to their performance status. The survival curves (upper part of figure) for the 14 patients in the present study and the 163 in the conventionally treated group with a performance status of grade 1 or 2 are clearly very similar (hazard ratio 1.03; 95% confidence interval 0.59–1.80). In marked contrast, those for the eight patients from the present study and 52 from the conventionally treated group with a performance status of grade 0 strongly suggest that with the intensive alternating regimen the long-term survival rate was higher (hazard ratio 0.38, 95% confidence interval 0.19–0.78).

### Discussion

We are aware of the difficulties inherent in subgroup analyses, particularly in non-randomised comparisons involving small patient numbers. Nevertheless, there is a strong suggestion from the present long-term follow-up that there is a real benefit from the alternating scheduling, but that it may be confined to the small subgroup of patients with WHO performance status of grade 0 at the time treatment is started. Indeed, all four of the patients still alive 1,274 days (42 months) or more after the start of treatment came from this subgroup, these being four of the total of eight in this subgroup.

The results for all 22 patients show that survival rates of

**Figure 1** Survival from date of start of treatment for all 22 patients in the present study (——) compared with the 215 patients with limited disease and performance status of WHO grade 0, 1 or 2 in a concurrent multicentre randomised MRC trial (——) involving conventionally scheduled chemotherapy and radiotherapy.

**Table I** Characteristics of the 22 patients with confirmed SCLC and the results of treatment. The patients are ranked according to the amount of chemotherapy (CT) and radiotherapy (RT) they received

| *Patient* | *Performance status on admission (WHO grade)* | *Number of courses of treatment received* CT | RT | *Duration of survival (days)*[a] | *Reason treatment not completed* | *Main cause of death* |
|---|---|---|---|---|---|---|
| *Cisplatin 100 mg m$^{-2}$* | | | | | | |
| 1 | 0 | 6 | 3 | 1,867 + | – | – |
| 2 | 0 | 5 | 3 | 1,598 | Toxicity | NSCLC in opposite lung |
| 3 | 1 | 5 | 3 | 275 | Toxicity | Cancer |
| 4 | 2 | 5 | 3 | 444 | Toxicity | Cancer |
| 5 | 1 | 4 | 3 | 333 | Toxicity | Cancer |
| 6 | 1 | 4 | 3 | 277 | Toxicity | Cancer |
| 7 | 1 | 3 | 3 | 808 | Toxicity | Cancer |
| 8 | 0 | 3 | 2 | 1,527 + | Toxicity | – |
| 9 | 0 | 1 | 1 | 308 | Progression | Cancer |
| 10 | 2 | 1 | 0 | 21 | Death | Bronchopneumonia |
| 11 | 1 | 1 | 0 | 4 | Death | Sudden collapse |
| 12 | 1 | 1 | 0 | 18 | Death | Bronchopneumonia |
| *Cisplatin 80 mg m$^{-2}$* | | | | | | |
| 13 | 1 | 6 | 3 | 1,295 | – | Unrelated, no cancer |
| 14 | 1 | 6 | 3 | 554 | – | Pulmonary embolism |
| 15 | 0 | 6 | 3 | 1,274 + | – | – |
| 16 | 0 | 6 | 3 | 1,440 + | – | – |
| 17 | 1 | 6 | 3 | 325 | – | Cancer |
| 18 | 2 | 5 | 3 | 248 | Toxicity | Cancer |
| 19 | 2 | 5 | 3 | 287 | Toxicity | Cancer |
| 20 | 1 | 4 | 2 | 172 | Progression | Cancer |
| 21 | 0 | 3 | 2 | 145 | Progression | Cancer |
| 24 | 0 | 2 | 0 | 37 | Death | Sudden collapse |

[a]+ indicates that the patient was still alive at the time of analysis. Patients 22 and 23 did not have SCLC.

**Figure 2** **a**, Survival from date of start of treatment for the 14 patients with performance status of WHO grade 1 or 2 in the present study (——) compared with the 163 similar patients with performance status of WHO grade 1 or 2 in a concurrent multicentre randomised MRC trial (——) involving conventionally scheduled chemotherapy and radiotherapy. **b**, Corresponding curves for the eight patients with performance status of WHO grade 0 in the present study and the 52 with performance status of WHO grade 0 in the randomised trial.

around 40% at 1 year, 30% at 2 years and 25% at 3 years can be achieved when patients with SCLC of limited extent and with good performance status are treated with an intensive regimen of alternating chemotherapy and radiotherapy. These findings confirm those of Arriagada and his colleagues, who reported a survival rate of 26% at 3 years in a study of 109 patients (Le Chevalier *et al.*, 1987), and of the Eastern Cooperative Oncology Group, which reported a progression-free survival rate of 33% at 3 years in a study of 34 patients (Johnson *et al.*, 1993).

Nevertheless, none of these phase II studies provides conclusive evidence of the superiority of alternating over conventional scheduling. Indeed, these promising findings could be the result of patient selection. The European Organization for Research and Treatment of Cancer (EORTC) is currently conducting a randomised trial with a planned intake of 360 patients in which a regimen of cyclophosphamide, doxorubicin and etoposide plus thoracic radiotherapy is being given either with conventional sequential scheduling, the radiotherapy being given after completion of the five cycles of chemotherapy, or with alternating scheduling (protocol 08877). As far as we are aware, this is the only randomised trial that is making the important comparison of alternating *vs* conventional scheduling. The results are therefore awaited with great interest, although the chemotherapy is not as intensive as that used in this study. It is also desirable that other randomised trials investigate this comparison further.

In the present study, four of the patients died within 6 weeks of starting treatment, and treatment was considered a contributory cause of death in all four (Bleehen *et al.*, 1991). This raises the possibility that the long-term results of treating SCLC might be improved if ways could be found of eliminating or greatly reducing the risk of early, treatment-related death (Morritu *et al.*, 1989; MRC Lung Cancer Working Party, 1993).

We would urge other groups to report the results of intensive treatment policies in patients with good performance status. Other reports (reviewed by Johnson *et al.*, 1993) have not shown results separately for patients of different performance status. It would be of great interest to see if similar patterns of survival according to performance status are seen in other studies. None, as far as we are aware, has been analysed and presented in the way reported here.

Alternating chemotherapy and radiotherapy is logistically demanding and carries a substantial risk of major toxicity, but if it can offer even a small subgroup of patients a better chance of cure it may be worth the risk.

### References

ARRIAGADA, R., LE CHEVALIER, T., RUFFIE, P., BALDEYROU, P., DE CREMOUX, H., MARTIN, M., CHOMY, P., CERRINA, M.L., PELLAE-COSSET, B., TARAYRE, M., SANCHO-GARNIER, H., THE GROP & THE FRENCH CANCER CENTER'S LUNG GROUP (1990). Alternating radiotherapy and chemotherapy in 173 consecutive patients with limited small cell lung carcinoma. *Int. J. Radiat. Oncol. Biol. Phys.*, **19**, 1135–1138.

BLEEHEN, N.M., GIRLING, D.J., GREGOR, A., LEONARD, R.C.F., MACHIN, D., MCKENZIE, C.G., MORGAN, D.A.L., SMYTH, J.F., SPITTLE, M.F., STEPHENS, R.J. & YOSEF, H.M.A. (1991). A Medical Research Council phase II trial of alternating chemotherapy and radiotherapy in small-cell lung cancer. *Br. J. Cancer*, **64**, 775–779.

JOHNSON, D.H., TURRISI, A.T., CHANG, A.Y., BLUM, R., BONOMI, P., ETTINGER, D. & WAGNER, H. (1993). Alternating chemotherapy and twice-daily thoracic radiotherapy in limited-stage small-cell lung cancer: a pilot study of the Eastern Cooperative Oncology Group. *J. Clin. Oncol.*, **11**, 879–884.

LE CHEVALIER, T., ARRIAGADA, R., BALDEYROU, P., DE CREMOUX, H., RUFFIE, P., MARTIN, M., CERRINA, M.L., DE THE, G., SANCHO-GARNIER, H. & HAYAT, M. (1987). Chemoradiotherapy combination in small cell lung carcinoma. Limits and results in 109 patients treated with an alternating schedule. *Bull. Cancer*, **74**, 559–564.

MEDICAL RESEARCH COUNCIL LUNG CANCER WORKING PARTY (1993). A randomised trial of 3 and 6 courses of etoposide cyclophosphamide methotrexate and vincristine or 6 courses of etoposide and ifosfamide in small cell lung cancer (SCLC). I. Survival and prognostic factors. *Br. J. Cancer*, **68**, 1150–1156.

MORRITU, L., EARL, H.M., SOUHAMI, R.L., ASH, C.M., TOBIAS, J.S., GEDDES, D.M., HARPER, P.G. & SPIRO, S.G. (1989). Patients at risk of chemotherapy-associated toxicity in small cell lung cancer. *Br. J. Cancer*, **59**, 801–804.

WORLD HEALTH ORGANIZATION (WHO) (1979). *WHO Handbook for Reporting Results of Cancer Treatment*, WHO Offset Publication No. 48. WHO: Geneva.